# Novel V-shaped kiss flap harvest technique for the forearm free flap in soft tissue reconstruction

**DOI:** 10.1186/s12957-023-02989-9

**Published:** 2023-04-10

**Authors:** Wenquan Zhao, Wenyuan Zhu, Dan Yu, Huiyong Zhu, Jianhua Liu, Youkang Ni

**Affiliations:** Department of Oral and Maxillofacial Surgery, The First Affiliated Hospital, College of Medicine, Zhejiang University, #79 Qingchun Road, Hangzhou, Zhejiang Province 310003 People’s Republic of China

**Keywords:** Forearm free flap,, Postoperative complications,, Esthetics,, Reconstructive surgical procedures,, Oral neoplasms

## Abstract

**Introduction:**

Radial forearm flap (RFF) is widely used in oral reconstruction. However, the donor-site defect remains the main limit. In this paper, V-shaped kiss RFF (VRFF) is described as a novel technique to improve aesthetics and function of it. A retrospective study was conducted to introduce VRFF and evaluate its effect and safety.

**Methods:**

A total of 21 patients who underwent VRFF for oral reconstruction, and 23 patients who underwent conventional RFF from February 2016 to April 2018 were included in this study. Direct comparisons were made on patient’s subjective evaluation of postoperative hand function and degree of scarring and objective donor-site function assessment including range of wrist movements and grip strength before and after surgery between the two groups.

**Results:**

No skin grafts were used in the VRFF group, and 20 of 21 patients achieved primary healing at donor site, while all patients from the RFF group had skin grafts. And 18 of 23 patients achieved primary healing. The postoperative scar score of donor site in the VRFF group was significantly higher than that in the RFF group (3.4 vs 2.8, *P* = 0.035). There were no significant differences in other subjective evaluation and donor-site morbidity and hand function assessment.

**Conclusion:**

VRFF is able to provide a new and simple method to close donor-site defect and realize a better healing in donor site.

## Introduction

Oral soft tissue defects significantly compromise patient aesthetics and function. Therefore, reconstruction of these defects restoring tissue anatomy and mobility are critical for patients. In 1981, Chinese scholar Yang Guo first introduced a radial forearm flap (RFF) in clinical reconstruction [1] . RFFs have numerous advantages over other bulkier alternatives, with a consistently high survival rate (90–100%) [2, 3]. It has become the workhorse for head and neck and perioral reconstruction [4, 5].

Closure of the donor site defect remains the main challenge in RFF. Several methods have been proposed to close donor-site defects, including cross-suturing [6], ulnar forearm perforator flap [7], artificial dermis graft [8], tissue expansion [9], hinged forearm split-thickness skin graft [10], local hatchet flap [11], local full-thickness skin graft [12], and snake design RFF [13], etc. These alternative methods provide references for future clinical work. However, they all present with a distinctive set of drawbacks, most commonly inferior aesthetics and steep surgical learning curves.

This work introduces and evaluates the effectiveness and safety of a new technique for closing RFF donor-site defects based on a V-shaped kiss radial forearm flap (VRFF). It is made of two connected islands that kiss each other; therefore, it was named the kiss flap.

## Materials and methods

We retrospectively reviewed patients who underwent oral cancer resection and RFF reconstruction in our department from February 2016 to April 2018. The inclusion criteria were as follows: (1) preoperative diagnosis of oral carcinoma, (2) age between 18 and 80 years, and (3) size of resultant oral defect deemed suitable for reconstruction with RFF. The exclusion criteria were as follows: (1) any vascular, nerve, or orthopedic lesions in the forearm and (2) a history of surgery and trauma in the forearm. Finally, we identified 44 patients who underwent RFF reconstruction, of whom 23 had conventional RFF, and 21 had VRFF.

The informed consent were obtained from patients. The ethics committee of The First Affiliated Hospital, Zhejiang University College of Medicine, approved the study (the number of approval protocol: (2021) IIT-326).

### VRFF flap design

All patients underwent Allen’s test preoperatively to ascertain the presence and patency of collateral circulation. All flaps were raised by the first author of this study simultaneously with tumor resection starting in the morning. After tumor resection, reconstruction of the resultant soft-tissue defect with VRFF was initiated.

VRFF flap was designed according to the size of soft tissue defects. The donor-site defect (the whole flap) should not exceed 6 cm in width and 8 cm in length.

**Fig. 1:**
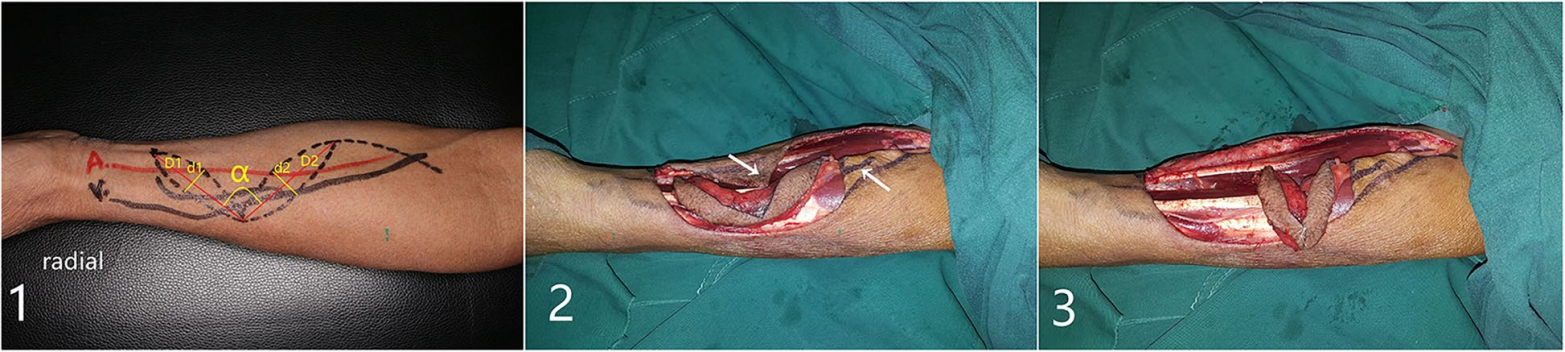
(**1**) Design of V-shape kiss radial forearm Flap. A: Radial artery; V: Cephalic vein; D1 = D2 = The length of recipient-site defect, D1 = D2 ≤ 8cm; α ≥ 90°; d1 + d2 = The width of recipient-site defect，d1 = d2 ≤ 3cm. (**2**) There are two skin angles in two sides. (**3**) Fold two flaps side to side

Figure 1-1 displays the schematic of the V-kiss flap design. The solid line indicates the course of the blood vessels. We can see that the flap contains a radial artery and a cephalic vein.

The dotted line indicates skin incision. All flaps were raised using a standard inflated tourniquet. The VRFF flap consists of two skin paddles connected by a bridge of the de-epithelialized tissue and skin. The bridge consists of feeding vessels and surrounding soft tissue, ensuring the vascularity of VRFF. The skin surface of the bridge can be disconnected if greater flap rotation is required. The two skin paddles form a V-shaped, with the apex facing the radial side of the cephalic vein, including the cephalic vein. The main arterial supply to the flap originates from the radial artery. The cephalic vein was the primary site of venous return. The long axes of the two flaps (D1 and D2) formed an obtuse angle. The lengths of D1 and D2 were the same and equal to the length of the soft tissue defect, which should be no more than 8 cm, usually 6 cm. The widths of the two flaps (d1 and d2) were the same, equating to half the width of the soft tissue defect, which should be no more than 3 cm. Since skin tension near the wrist is generally greater than that near the elbow, d1 can be slightly shorter than d2. The two fasciocutaneous flaps were harvested in the same manner as the conventional RFF.

**Fig. 2:**
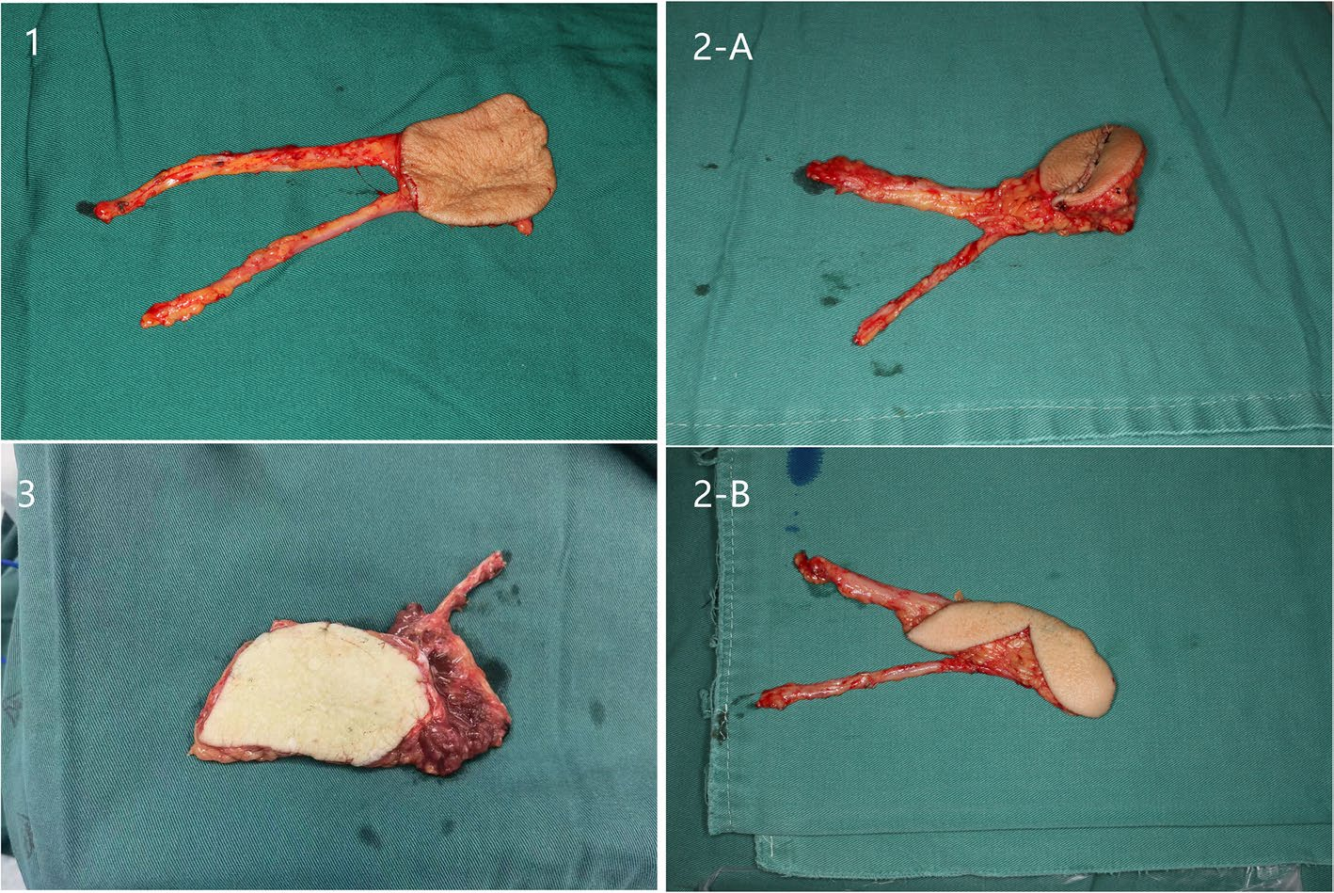
Different flaps are suitable for different ranges of tongue defect. (**1**) RFF; (**2-A**) VRFF; (**2-B**) unsutured VRFF; (**3**) ALT flap

The difference between VRFF and conventional RFF is that the skin between the two skin paddles was raised from the subcutaneous fat layer of the flap. When incising the skin, the width of the skin is less than the width of the de-epithelialized issue in the bridge (Fig. 2-2B). The skin is left in the original position of forearm area to directly close donor-site defect (the two arrows in Fig. 1-2 represent the skin). The de-epithelialized tissue below it, the same as the conventional RFF, is included in the flap. The de-epithelialized tissue was wider than the skin in the bridge, including the radial artery and cephalic vein, to ensure blood supply.

**Fig. 3:**
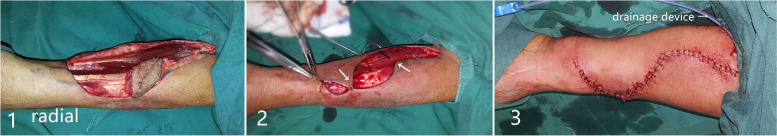
(**1**) Suture the two independent small flaps as VRFF (**2**) The closure of donor defect: directly suturing (**3**) Primary closure of donor defect

**Fig. 4:**
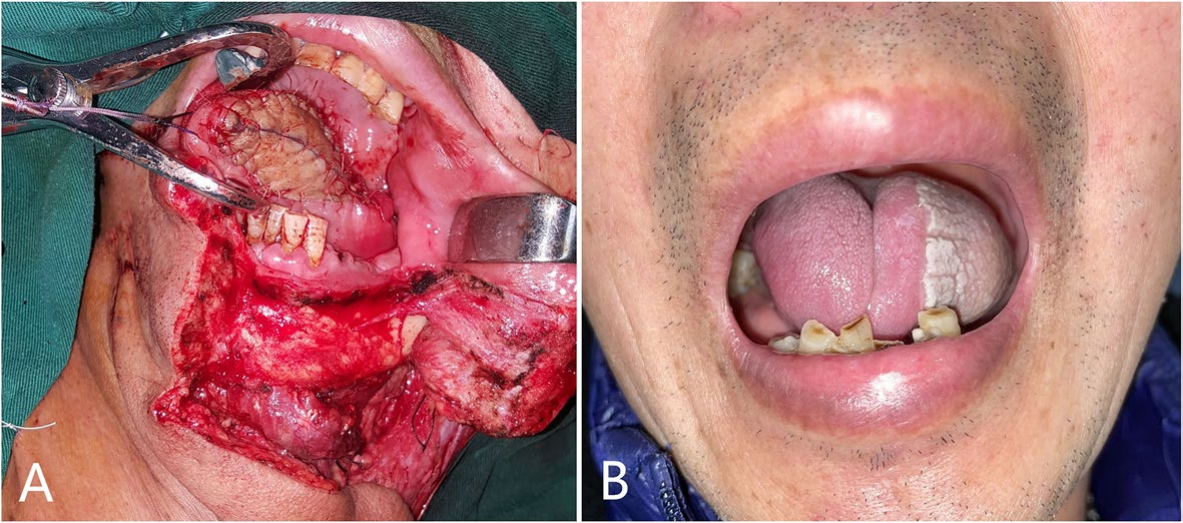
(**A**) The VRFF repairs tongue defect (**B**) a long-term photo of the same patient (30 months)

The main distinction lies in the donor site handling after flap harvesting. The surrounding skin is raised by undermining the supramuscular fascia. There were one angle on both sides (Fig. 1-2). The skin was used to close the wound to the donor site of the forearm. A part of the subcutaneous tissue, along with the radial artery/vein and cephalic vein, was contained in the forearm flap to ensure better blood supply. We extended the incision from the forearm midline to the fossa cubitalia at the ulnar tip of the small upper flap to expose the radial artery/vein and cephalic vein. The length of the flap pedicle can be increased as required. The other harvesting procedures were similar to those used for the conventional forearm flap. The two flaps were folded side-to-side and sutured on the one side to form a whole flap (Figs 1-3, 2-2A and 3-1). The vascular pedicle under the flap was protected by thick subcutaneous tissue. After suturing the flaps side by side, the vascular pedicle appeared as round and blunt contortions instead of sharp contortions. Moreover, it provided no hindrance to the arterial supply or venous return. The long axis of the flap was the long axis of the defect after tumor resection, and the short axis was the short axis of the defect area (Figs. 3-1 and 4-A). Once VRFF was harvested, the tourniquet was released. Based on the size, any small vessel other than VRFF pedicle should be cauterized or ligated, after which VRFF pedicle was divided. The flap was then transferred to the recipient site (Fig. 4-A). We examined the blood flow of the anastomotic vessels and used VRFF to close the oral soft tissue defect.

### Closure of donor site

Once VRFF pedicle was dissected, the donor-site defect was closed by the released skin, and the subcutaneous plane contributed to a decrease in the width of the wound. If excessive skin tension still exists, we will release the skin layer from the subcutaneous tissue so that the surrounding skin can be extended enough to be sutured directly. One skin angle was drawn to the other side and sutured (Fig. 3-2 and 3-3). A drain was placed on the wound (Fig. 3-3). In conventional RFF, a full-thickness skin graft from the axilla is used to close the donor site defect, and the skin graft surface is covered with reversing pressure dressing. Additionally, the second donor site must be sutured directly.

#### Evaluation projects

We designed a scoring system for subjective evaluations consisting of donor-site scarring and wrist function scores. The visual analog scale (VAS) was used for reference and simplifying. Depending on the practical situation, the scores range from 1 to 5. The higher the score, the better was the patient’s satisfaction. The patients were asked to complete the evaluation scale postoperatively to evaluate the appearance and functional recovery of the donor site. An independent observer carried out the subjective evaluation 9 months after the operation.

**Fig. 5:**
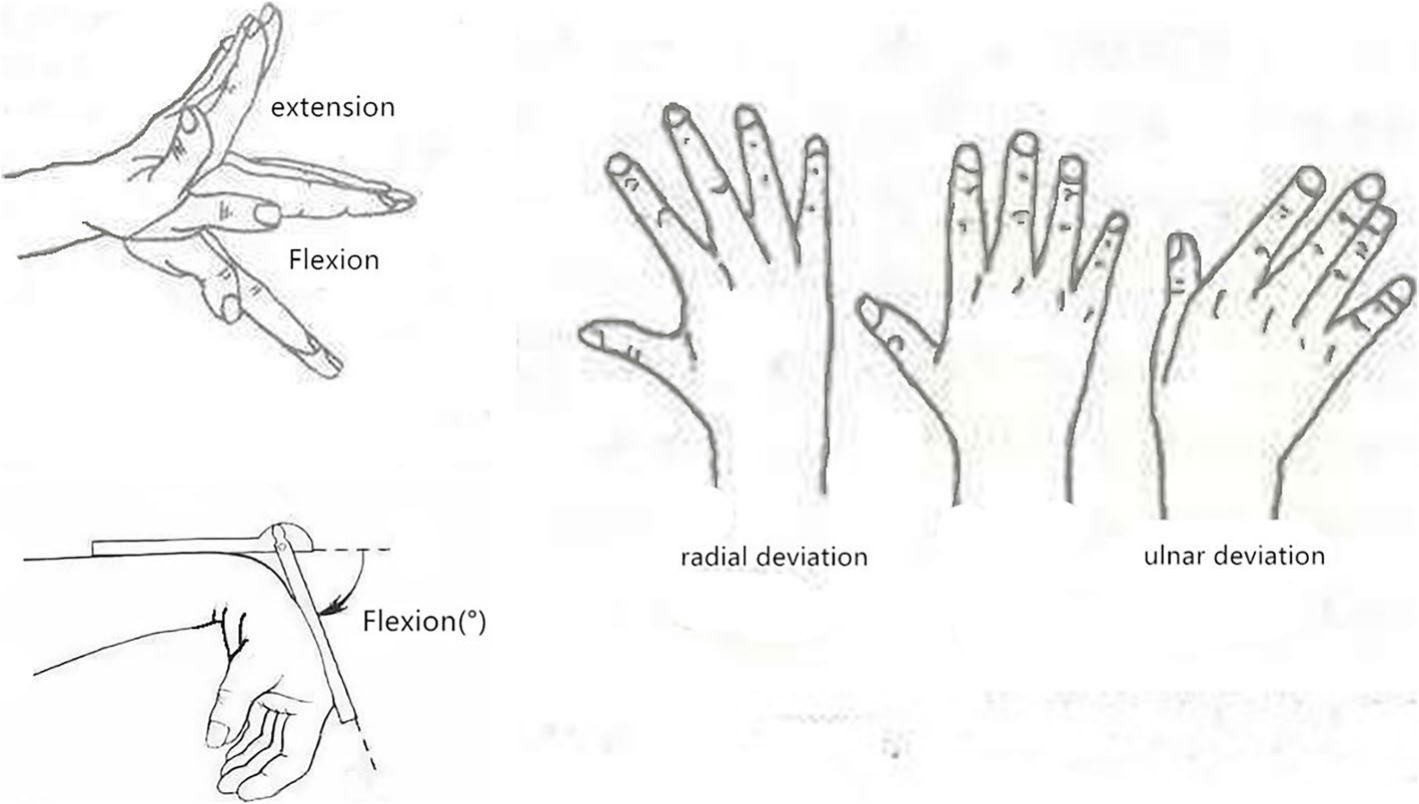
Range of wrist movements (ROMs)

Objective evaluation included a preoperative and postoperative range of wrist movements (ROMs) (flexion, extension, radial deviation, and ulnar deviation, Fig. 5) and grip strength. ROMs were measured with an angle meter, and grip strength was measured with an electronic grip dynamometer. Another independent measurer, who was blinded to the patient’s flap grouping, conducted the evaluation session both preoperatively and at 9 months postoperatively.

The flap harvesting and closing times of the donor site in the two groups were recorded.

### Follow-up

**Fig. 6:**
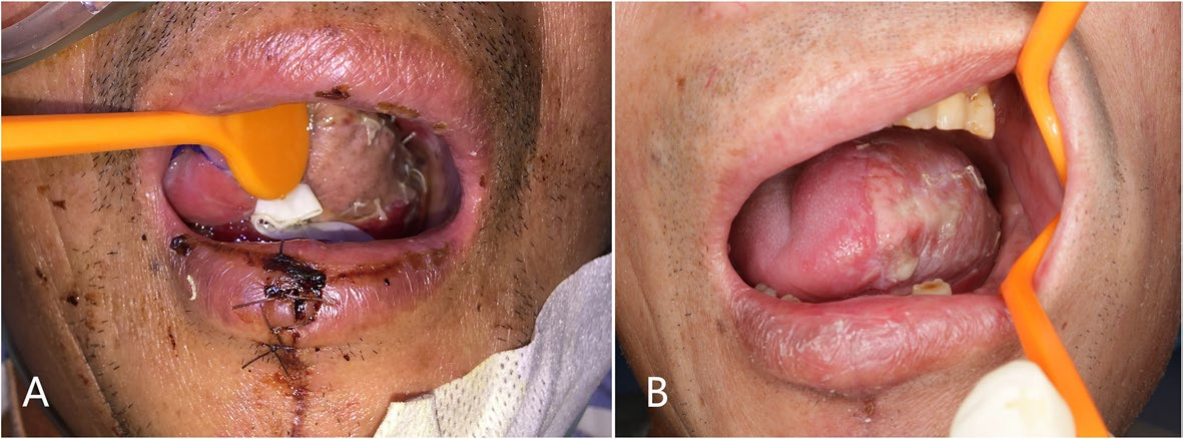
(**A**) The initial intraoperative flap insetting (**B**) Postoperation oral image

**Fig. 7:**
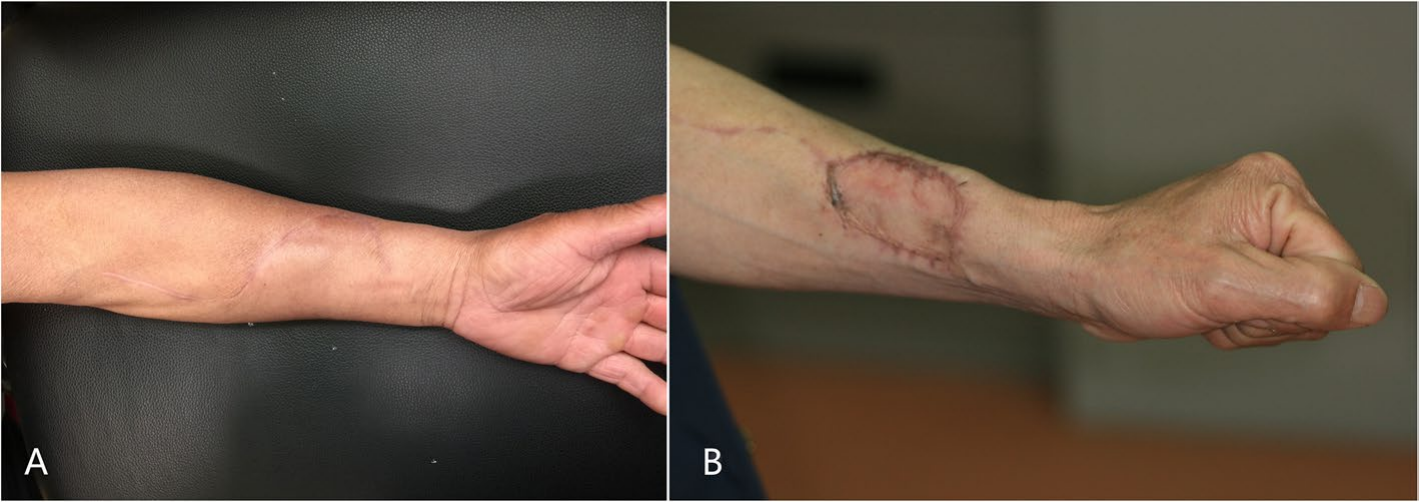
(**A**) The donor-site scar of VRFF at 9 months after operation (**B**) The donor-site scar of RFF at 9 months after operation

The follow-up of patients was conducted 1, 3, 6, 9, 12, 18, 24, and 30 months after the operation to check the growth of flap, scar, and donor-site function, and the donor-site morbidity was recorded (see Figs. 4-B, 6 and 7).

### Statistical method

SPSS 22.0 was used to analyze the datas. Mann–Whitney *U* test and the independent sample *T* test were adopted. *P* < 0.05 was considered as statistically significant.

## Results

In total, 21 patients received VRFF flaps, whereas 23 received conventional RFF flaps (see Table 1). In the VRFF group, donor site defects were closed primarily without additional skin grafts. In the RFF group, donor site defects were closed with axial full-thickness skin grafts.

**Table 1:** General demographic information of the VRFF group

No	Age (years)	Gender (F/M)	TNM	Tumor location	Pathology	Defect size (cm × cm)	Follow-up(m)
1	63	M	T_1_N_0_M_0_	Buccal mucosa	SCC	3.5 × 4.5	30
2	44	M	T_2_N_0_M_0_	Tongue	SCC	4.5 × 5.0	29
3	53	M	T_2_N_1_M_0_	Tongue	SCC	4.5 × 5.5	28
4	58	M	T_2_N_0_M_0_	Tongue	SCC	4.0 × 5.5	27
5	44	F	T_3_N_1_M_0_	Tongue	SCC	5.0 × 6.5	26
6	41	M	T_1_N_0_M_0_	Buccal mucosa	SCC	3.5 × 4.0	25
7	67	M	T_2_N_1_M_0_	Tongue	SCC	4.5 × 5.0	24
8	45	F	T_2_N_0_M_0_	Tongue	SCC	4.0 × 5.0	24
9	65	F	T_2_N_0_M_0_	Tongue	SCC	4.0 × 5.5	22
10	59	M	T_2_N_0_M_0_	Buccal mucosa	SCC	3.5 × 4.0	21
11	66	M	T_1_N_0_M_0_	Buccal mucosa	SCC	3.5 × 4.0	20
12	51	F	T_2_N_0_M_0_	Tongue	SCC	4.5 × 5.0	19
13	46	M	T_1_N_0_M_0_	Tongue	SCC	3.5 × 4.5	19
14	55	F	T_2_N_0_M_0_	Tongue	SCC	4.0 × 5.0	18
15	64	F	T_1_N_0_M_0_	Buccal mucosa	SCC	3.5 × 4.5	18
16	59	M	T_2_N_0_M_0_	Tongue	SCC	4.5 × 5.5	17
17	69	M	T_1_N_0_M_0_	Tongue	SCC	3.5 × 4.0	17
18	49	M	T_2_N_1_M_0_	Buccal mucosa	SCC	4.5 × 5.0	15
19	61	M	T_2_N_0_M_0_	Tongue	SCC	4.5 × 5.5	15
20	56	M	T_1_N_0_M_0_	Tongue	SCC	3.5 × 4.0	14
21	55	M	T_1_N_0_M_0_	Tongue	SCC	3.5 × 4.0	14

There was one case of a venous crisis in the VRFF group and RFF group, respectively. All flaps ultimately survived. There was one case of partial flap necrosis in the VRFF group and 2 cases in the RFF group.

All flaps were raised from the left forearm and non-dominant side. The mean flap harvesting time in the VRFF group was slightly longer than in the RFF group (50.71 ± 6.99 min vs 46.61 ± 7.23 min, *P* = 0.062). For donor-site defect closing time, the VRFF group was significantly shorter at 17.05 ± 2.42 min, compared to 24.57 ± 5.35 min for RFF group (*P* < 0.001). This is exclusive of the time required to raise the additional skin graft.

**Fig. 8:**
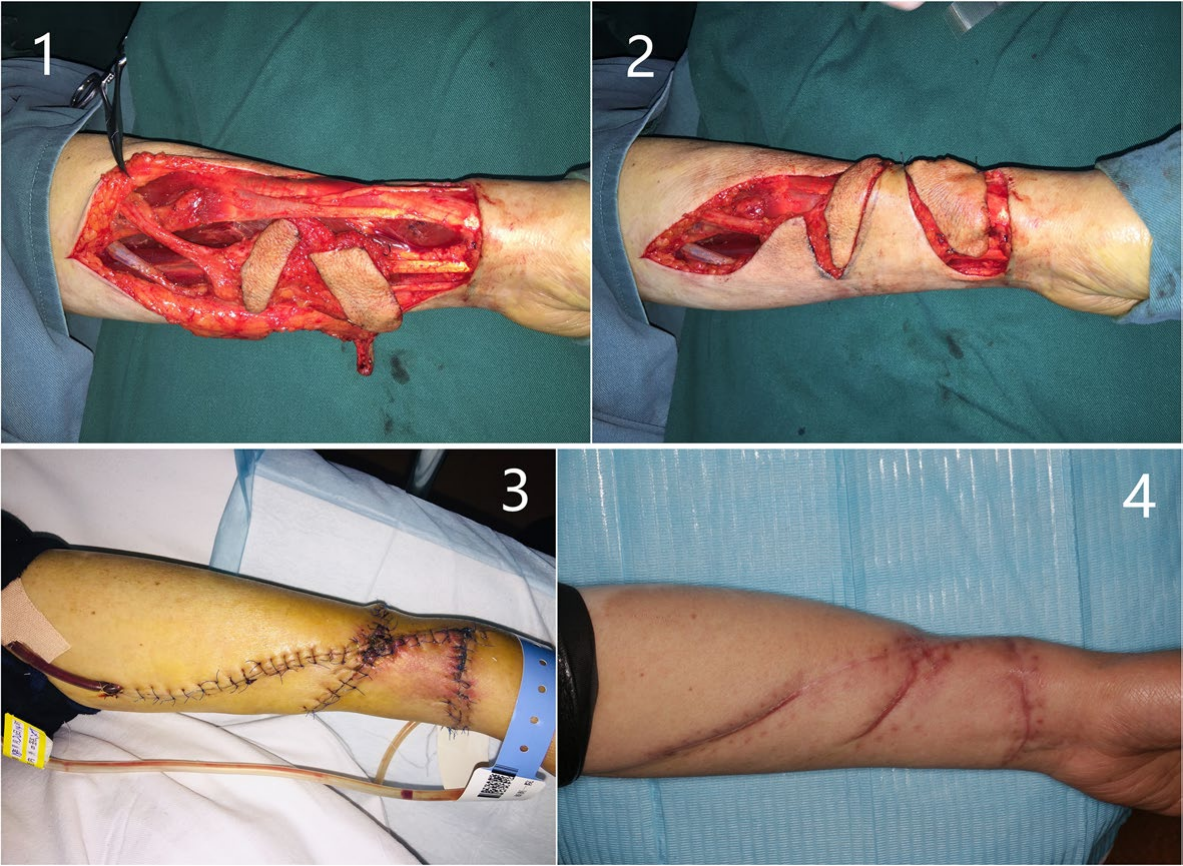
Patients required additional sliding flap repair due to excessive local wound tension and reach Primary healing

Regarding donor-site morbidity, there was one patient with mild tendon adhesion in the VRFF group, while two patients required additional sliding flap repair due to excessive local wound tension (see Fig. 8). One patient experienced local wound rupture when stitches were removed 10 days after surgery, which was promptly repaired and healed uneventfully afterward. The donor-site scar was not obvious in any of the patients in the VRFF group. 20/21 patients achieved primary healing (see Fig. 7-A). In the RFF group, one patient also experienced tendon adhesions. All patients were treated with free skin grafting to close the defects, among which five patients experienced partial skin necrosis in varying degrees. After several sessions of conservative wound dressings, the necrotic sites eventually closed, leaving conspicuous scarring and skin tension. Only 18/23 patients achieved primary healing (see Fig. 7-B).

Four patients in the VRFF group and six in the RFF group reported numbness on the dorsum of the thumb and the lateral and palmar aspects of the thenar eminence. However, the numbness was mild and spontaneously improved over time. Other commonly reported morbidities, such as cold intolerance, were not encountered in this study. No obvious decline in hand function was observed in either of the groups.

### Subjective evaluation

Regarding the function and appearance of the donor site, the results demonstrated that patient satisfaction with donor-site scarring was significantly higher in the VRFF group than in the RFF group, 3.4 vs 2.8, *P* = 0.035 (see Tables 2 and 3). There was no statistically significant difference in the donor function scores between the two groups, *P* = 0.970.

**Table 2:** Scar of donor site

Score	VRFF (*n* = 21)	RFF (*n* = 23)
1	1	2
2	2	6
3	7	10
4	9	4
5	2	1
Total	21	23
Mean score	3.4	2.8

**Table 3:** function of donor site

Score	VRFF (*n* = 21)	RFF (*n* = 23)
1	0	0
2	2	1
3	5	7
4	9	10
5	5	5
Total	21	23
Mean score	3.8	3.8

### Objective evaluation

Finally, 16 patients in the VRFF and RFF groups underwent preoperative and postoperative assessments of ROMs and grip strength. The remaining patients in the study either declined functional assessment or were lost to follow-up. The results are presented in Table 4. No statistically significant differences were found between the two groups regarding ROMs and strength (insert data or *P* value to support this statement).

**Table 4:** ROMs of wrist and grip strength between the VRFF group and RFF group

		VRFF (*n* = 16)	RFF (*n* = 16)	*P*
Preoperation				
ROMs	Flexion (°)	71.1 ± 17.7 54.1 ± 11.9	73.1 ± 11.1 54.4 ± 9.7	.694 .94
	Extension (°)			
	Ulnar deviation (°)	43.7 ± 6.1 20.1 ± 9	45.2 ± 6.8 21.4 ± 10.8	.731 .52
	Radial deviation (°)			
Grip strength (kg)		22.3 ± 10.8	26.5 ± 10.3	.266
Postoperation				
ROMs	Flexion (°)	65.1 ± 17.6 53.4 ± 12.7	68.9 ± 14.6 51.4 ± 13.2	.505 .679
	Extension (°)			
	Ulnar deviation (°)	43.8 ± 9 18.3 ± 10	43.6 ± 7.5 18.6 ± 12.7	.945 .948
	Radial deviation (°)			
Grip strength (kg)		20.9 ± 10.8	22.8 ± 10.1	.613

ROMs and grip strength measurements in the VRFF group before and after surgery are illustrated in Table 5. Although it can be observed that ROMs and grip strength both decreased after operation, the differences were not statistically significant (insert data or *P* value to support the statement).

**Table 5:** ROMs of wrist and grip strength in the VRFF group

		Preoperation	Postoperation	*P*
ROMs	Flexion (°)	71.1 ± 17.7 54.1 ± 11.9	65.1 ± 17.6 53.4 ± 12.7	.114 .712
	Extension (°)			
	Ulnar deviation (°)	43.7 ± 6.1 20.1 ± 9	43.8 ± 9 18.3 ± 10	.464 .971
	Radial deviation (°)			
Grip strength (kg)		22.3 ± 10.8	20.9 ± 10.8	.445

## Discussion

The closure of donor site defects remains one of the main challenges of conventional RFF. A separate skin graft is usually required to close the defect, thus creating a second donor-site defect. Skin grafts are prone to scar formation, poor donor-recipient skin shade matching, and additional complications from a second donor site. In addition, skin graft failure may lead to delayed wound healing and tendon exposure in the forearm. Many studies have reported new approaches for closing donor site defects. Moazzam et al. used cross-suturing to narrow the donor site defect size and close with smaller skin grafts [6]. All patients were satisfied with their appearance. However, skin grafts are required. Hsieh et al. used a bilobed flap. The larger one was used to repair the donor-site defect, and the smaller one was used for the defect of the larger one [7]. No patient satisfaction was noted in this study. The main disadvantage is the long postoperative scar that results from multiple curved incisions and tissue rotation. Lee preferred an artificial dermis. Donor-site scars are less visible without complaints from the patient [8]. However, an artificial dermis is costly, with a limited supply. Bonaparte et al. reported on the use of tissue expansion. However, only four cases (33.3%) had their donor site closed primarily [9]. Other cases have indicated that redundant skin grafts are required. The aesthetics scores of patients in the treatment group were not higher than those in the control group. Boahene et al. reported a hinged forearm split-thickness skin graft (SGST), and the RFF can be harvested under it [10]. However, no patient satisfaction was reported. We believe that large STSG are difficult to harvest. Due to the lack of dermal coverage, the integrity and healing of RFF may be compromised. Lane used a hatchet-like flap to extend the incision and raise the skin to close the defect [11]. The Vancouver Scar Scale (VSS) and visual analog scale (VAS) produced positive results. However, this method is not recommended for large donor-site defects. Riecke adopted two pieces of local full-thickness skin graft [12]. No patient reported cold intolerance or complaints of poor aesthetic results. We believe that the possibility of skin graft failure is higher when comparing two pieces of local full-thickness skin grafts with one piece of the skin graft from the second donor site. Garg proposed a snake-like RFF design that contributed to subsequent primary closure [13]. The snake-like RFF was folded side-to-side when repairing the oral defects. Patient satisfaction was not observed. The main disadvantage of this design is that it limits the length of the pedicle and degree of skin paddle rotation.

The present study introduced a new technique to improve the ease of donor-site closure and the final aesthetic outcome. Twenty patients receiving VRFF treatment achieved primary skin healing with inconspicuous scarring. VRFF have also demonstrated consistent clinical efficacy and patient safety. As for the time of flap harvesting, no statistically significant difference was found between VRFF and conventional RFF, which suggests that despite the modified design of two island flaps, VRFF required no additional time to raise and transfer. As for the closure time of donor site, the procedures of conventional RFF are more complex, including skin extraction and suturing at the second donor site, trimming and thinning of the skin graft, and reversal of pressure dressing to repair the donor-site defect. Moreover, a skin graft from an additional donor site and closure of the second donor site were not required. Therefore, the closure time of the donor site was significantly shorter than that in the RFF group. In this study, the results verified that the VRFF group had a significantly shorter donor-site defect closing time, 17.05 ± 2.42 min vs 24.57 ± 5.35 min, *P* < 0.001. The total time of donor site surgery was also shorter in the VRFF group than in the RFF group. Evidently, VRFF significantly reduces the flap harvesting operation time and simplifies surgical procedures, which would likely contribute to a faster and smoother recovery for the patient.

Except for scars and limited wrist function, there were no significant clinical complications or patient complaints related to donor-site morbidities and function during the follow-up period. Postoperative donor-site morbidity has been well-documented in the literature [14, 15]. However, forearm flap donor-site morbidities are relatively minor. Flap harvesting and donor-site defect closure had a slight influence on hand function in this study and many other studies [16–18].

In our objective evaluation, there was no significant difference between the two groups in terms of the effect of flap harvesting on postoperative hand function. This suggests that VRFF flap is a safe alternative to the traditional RFF flap. Compared with the VRFF group, a heavier scar contracture at the donor site in the RFF group after receiving free skin grafts may cause a more limited range of motion. This also reflected the functional improvement of VRFF flap. However, both VRFF and RFF flaps have some effects on hand function. Many other studies on donor-site function have been reported. Gravvanis performed a functional analysis of the hands after the donor site was closed using artificial skin grafts [19]. They found no significant differences between preoperative and postoperative outcomes. Byun et al. also reported similar outcomes [17]. Lane used a local hatchet flap and found that the grip strength of the surgical side hand was significantly reduced by 5 kg compared with that of the non-surgical side hand [11]. Lutz found a significant difference in preoperative and postoperative grip strength after skin grafting at RFF’s donor site [18]. Riecke reported a 2.7 kg reduction in grip strength, a 2.1 kg reduction in tip pinch strength, and a 12.5° reduction in dorsal extension after RFF harvesting when a local full-thickness skin graft was used to close the primary donor site [12]. A prospective study by Mashrah found a significant difference between preoperative and postoperative wrist extension and a significant reduction in grip strength (3.68 kg when using a local bilobed flap) [4].

VRFF is applicable for most cases indicated for RFF repair, and it is much more suitable for patients with laxed forearm skin. In our experience, VRFF flap is particularly useful for tongue defects because of its ridgy shape. For buccal defects, the conventional RFF flap design fits better because of its flat shape. Tongue defects are the most common maxillofacial defects. When repairing a tongue defect, many factors are involved in flap selection, such as the volume (defect and flap volume), shape, and pedicle length of the potential donor flap. RFF, VRFF, and ALT flaps are suitable for different ranges of tongue defects. ALT flap is used for larger tongue defects, such as hemi- and total-glossectomy defects. Compared to the flat shape of conventional RFF, the two sutured island flaps of VRFF provide thicker tissue bulges with enhanced stereoperception. The flap was thicker in the middle and thinner at both ends, which can better restore the native shape of the tongue after substantial resection. In Fig. 2, we show the differences between the three flaps. This requires a relatively long pedicle for tongue reconstruction. All anastomotic vessels in the VRFF group were selected as facial arteries, which were better for anastomosis than the superior thyroid arteries. The facial arteries were closer to the oral defects and required only a 5–6-cm vascular pedicle. In addition, the soft tissue of the pedicle has a certain ductility, so the length of VRFF vascular pedicle could still meet the anastomosis requirement when repairing the tongue tissue. In the last part of this article, we discuss the vascular pedicle problem at the last part of the article.

The recommended consideration for VRFF design and harvest are as follows:

- 1. The size of the donor-site defect should be no more than 6 cm in width and 8 cm in length. The maximum width of each individual island flap should be kept within 3 cm; otherwise, subsequent primary closure of donor site wound is difficult. If the surrounding skin is too tight, the island flap size should be reduced accordingly. Alternatively, additional local sliding or transposition flaps can be incorporated to facilitate wound closure. Considering the varying degrees of skin relaxation at different sites, the flap width near the elbow can be slightly larger than that near the wrist.
- 2. The angle between the two flaps should not be too small, and the obtuse angle is appropriate. The entire VRFF is located on the radial side. Being close to the radial side allowed the wound to be more hidden. The obtuse angle also increases skin relaxation at the incision site, which is conducive to tension-free skin closure.

The advantages of VRFF flap are obvious. It can achieve simplified primary wound closure without needing an additional skin graft, achieving uneventful healing without significant complications and good functional and aesthetic results at the donor site after flap harvesting. It avoids the potential issues of graft skin color mismatch, scar contracture, pain, and infection risk associated with the second donor site. The harvesting procedure was relatively simple. The disadvantage is the limited flap size, which is also an issue with the conventional RFF. The apex of the flap may be at a higher risk of partial necrosis.

The blood supply and vascularity length have been of concern because of the longer vessel pedicle sacrificing in VRFFs and contortions after suturing the flap side by side. Compared with the conventional RFF, VRFF lacks the skin tissue of V-triangle area between the two skin paddles. It contains the same subcutaneous tissue and blood vessels as conventional RFF. Therefore, the perforator branch at the distal 1/3 of the arm was still completely retained in the flap, supplying blood to the two skin paddles. Therefore, the blood supply was sufficient. According to our observations, there is no kinking of the pedicle in VRFF after suturing the two skin paddles side by side. The vascular pedicle under the flap was protected by thick subcutaneous tissue. After suturing, the vascular pedicle appeared as round and blunt contortions instead of sharp contortions. Therefore, there would be no problems such as blood supply disturbances caused by kinks. Intraoperatively, we also observed no obvious abnormality in venous return velocity.

Regarding the length of the vessel pedicle, the accessible pedicle length of VRFF is indeed 4–6 cm shorter than that of the traditional flap. The length of the radial artery that can be harvested is quite abundant, approximately 10–15 cm, even longer. The facial artery was used clinically as the recipient artery. The distance from the anastomotic stoma to the oral defect was shorter; therefore, VRFF pedicles must be too long. In our case, we did not encounter an insufficient pedicle. For some special cases, such as anastomosis that must be placed on the opposite side and a longer vessel pedicle is required, the conventional flap is recommended. In future studies, we will include the vascular pedicle length as the observation index.

In VRFF flap, the cephalic vein was still the main venous return site, and there were two points to protect the cephalic vein.

- 1. When the flap was raised, the entire flap was slightly inclined to the radial side. The ulnar edge can wrap around the radial artery and its perforator. The cephalic vein was closer to the center of the flap.
- 2. Compared with conventional RFF, VRFF lacks V-triangle skin tissue between the two skin paddles. VRFF retains the same subcutaneous tissue and blood vessels, including the perforator, in the flap, as in conventional RFF. Due to the larger angle between the two skin paddles, the length of the cephalic vein around the flap and the surrounding soft tissue are still similar to those of conventional RFF.

Therefore, the passage of blood back to the cephalic vein through the subcutaneous tissue was smooth, and the cephalic vein was used as the main return vein in our cases. In the future, if cephalic vein reflux is not smooth, it can also be remedied by the radial vein; however, this is rare.

The present study had some limitations. This was a retrospective study with a relatively small sample size and a short follow-up period. We are currently planning a prospective randomized controlled trial with a larger patient pool to substantiate the results of this study.

## Conclusion

VRFF has demonstrated a superior aesthetic outcome with simpler wound closure, shorter surgical time, predictable flap survival, and favorable patient satisfaction in recovery. Its impact on postoperative donor hand function and complications are comparable to that of the conventional RFF flap while eliminating the need for additional skin grafts for wound closure. Consequently, it can be considered a safe and effective alternative to conventional RFF flaps in clinical practice.

## Data Availability

The datasets generated and/or analyzed during the current study are not publicly available but are available from the corresponding author on reasonable request.
